# Interaction Between Race, Ethnicity, Severe Mental Illness, and Cardiovascular Disease

**DOI:** 10.1161/JAHA.121.025621

**Published:** 2022-06-14

**Authors:** Kevin O'Gallagher, James TH. Teo, Ajay M. Shah, Fiona Gaughran

**Affiliations:** ^1^ British Heart Foundation Centre of Research Excellence King’s College London London United Kingdom; ^2^ King’s College Hospital NHS Foundation Trust London United Kingdom; ^3^ Institute of Psychiatry, Psychology and Neuroscience King’s College London London United Kingdom; ^4^ South London and Maudsley NHS Foundation Trust London United Kingdom

**Keywords:** bipolar disorder, critical pathways, ethnicity, life expectancy, mental disorders, schizophrenia, Disparities, Revascularization, Treatment

## Abstract

Severe mental illnesses, such as schizophrenia or bipolar disorder, affect ≈1% of the population who, as a group, experience significant disadvantage in terms of physical health and reduced life expectancy. In this review, we explore the interaction between race, ethnicity, severe mental illness, and cardiovascular disease, with a focus on cardiovascular care pathways. Finally, we discuss strategies to investigate and address disparities in cardiovascular care for patients with severe mental illness.

**Nonstandard Abbreviations and Acronyms**
SMI: severe mental illness

Mental health disorders are a common and significant health care problem,^1^ with a global lifetime prevalence of up to 85% (in a predominantly White sample).^2^ Approximately 1% of the UK population has a diagnosis of a “severe mental illness” (SMI), such as schizophrenia or bipolar disorder.^3^ A diagnosis of mental illness more than doubles the risk of developing subsequent physical diseases at a younger age, with the risk of death at a younger age almost 4 times higher than in those without a mental disorder.^4^ This effect is magnified in people with SMI in whom significant morbidity and mortality is evident.^5^, ^6^ The number one cause of death in patients with SMI is not suicide or the primary psychiatric disease, but rather cardiovascular disease (CVD).^5^, ^6^ In addition, CVD is responsible for a larger overall loss in life expectancy across the population with SMI than suicide,^7^ with up to two thirds of deaths being attributed to preventable CVD.^6^ Increased risk of CVD in SMI is observed in both sexes and across all adult age groups.^5^ Moreover, although in the general population, rates of CVD, cardiovascular death, and modifiable risk factors have decreased steadily over the past few decades, this trend to improvement has not been matched in patients with SMI and therefore a mortality gap persists.^7^, ^8^ Currently in patients with SMI, rates of modifiable cardiovascular risk factors are high, whereas use of evidence‐based therapies is low.^9^, ^10^ The American Heart Association recognizes that SMI predisposes young patients to accelerated atherosclerosis and early CVD.^11^ People with bipolar disorder develop CVD over a decade earlier than nonpsychiatric controls (and with a standardized mortality rate of up to 8).^12^

It has been repeatedly reported that the prevalence of SMI varies between racial and ethnic groups and is affected by migration.^13^, ^14^, ^15^, ^16^ For example, in 2 separate observational studies of patients with first episode psychosis in London, UK, higher incidence rates were reported among most self‐ascribed racial and ethnic groups compared with the White British group, with this most marked in people of self‐ascribed Black African and Black Caribbean race and ethnicity, although interestingly this gap appears to be narrowing over time, with an increase in incidence in the White British group and some reduction in the relative risk for Black Caribbean men.^17^, ^18^ Of note, however, this is a phenomenon that is not worldwide, with limited epidemiological work on rates of psychosis in the Global South and the existing work not showing the large excess risk seen in people from underrepresented racial and ethnic groups as in large Western settings.^19^

A diagnosis of SMI confers an increased risk of all‐cause mortality across racial and ethnic groups: In a longitudinal cohort study of 18 201 people with SMI (defined as schizophrenia, schizoaffective disorder, and bipolar affective disorders) in the UK over 8 years, there was an increase in all‐cause mortality across all self‐ascribed racial and ethnic groups; however, Black African and Black Caribbean patients had decreased mortality relative to White British patients, with a similar trend seen in South Asian patients compared with White British patients.^20^ Likewise, in this study, all racial and ethnic groups demonstrated an increase in mortality from cardiovascular and cerebrovascular disease; the highest standardized mortality ratio was in Black African patients (3.85 [95% CI, 2.71–5.31]); however, the number of events was small and the standardized mortality ratio was not significantly different to the increased mortality seen in other racial and ethnic groups. In a larger US national retrospective cohort study (n=1 138 853) of patients with schizophrenia, all had increased mortality but with significant variation between (Medicaid‐defined) races and ethnicities: the Black non‐Hispanic group had a standardized mortality ratio less than half that of the White non‐Hispanic group (2.0 [95% CI, 2.0–2.0] versus 4.3 [95% CI, 4.6–4.7]).^5^ It is striking that there is significant excess cardiovascular mortality even in young adults with schizophrenia who otherwise would be considered at low risk of CVD: the standardized mortality ratio in the 20‐ to 34‐year‐old group was 4.5 (95% CI, 4.1–4.8), with similar standardized mortality for ischemic and nonischemic CVD.

## Social Determinants of Health Care Inequity

Race and ethnicity are social constructs and, as such, health disparities experienced by underrepresented racial and ethnic groups must be considered through the prism of social determinants of health, including structural racism.^21^, ^22^ Furthermore, when analyzing the relationship between SMI and race and ethnicity, we must consider the well‐documented disparities in cardiovascular health experienced by patients from underrepresented racial and ethnic groups.^23^ There are also clearly documented effects of social factors on the risks of developing SMI, including unemployment, social isolation, employment achievement, and evidence of neighborhood‐level variation, with effects consistent with the classic sociological models of mental disorders.^24^, ^25^

Stemming from the “double disadvantage hypothesis,” which predicts that multiple disadvantaged statuses interact to drive worse health status,^26^ it has been proposed that patients from underrepresented racial and ethnic groups with a diagnosis of SMI experience a “double jeopardy,” whereby SMI combines with factors relevant to race‐ and ethnicity‐related health disparities to drive physical disease.^27^ Furthermore, given that many of the adverse health effects experienced by patients with SMI are not driven solely by classic factors, such as race and ethnicity or social deprivation, it has been suggested that an SMI should be designated as a health disparity population in and of itself to both improve awareness and increase directed research.^28^

The finding that patients from underrepresented racial and ethnic groups with SMI have a lower adjusted mortality from CVD raises the question of why? In a study from a racially and ethnically diverse population in London, UK, it was found that although measures of social deprivation, urbanicity, and social fragmentation were associated with mortality risk in SMI, the “ethnic density” of an area (the proportion of residents from underrepresented racial and ethnic groups) was most strongly associated with all‐cause mortality: in the highest ethnic density areas, the relative risk of death reduced to approximately half of that of White British individuals.^29^ It is therefore clear that strong social factors are at play in the complex interaction between SMI, risk of death, and race and ethnicity. It is also apparent that the relationship of SMI to CVD is not simply linear, but rather a result of the interplay between a constellation of disparities in which the individual factors can have multiplicative, rather than simply additive, effects on outcome.

## Cardiovascular Risk Factors

CVD is known to be driven by a set of conditions and/or behaviors that are collectively referred to as “cardiovascular risk factors.” One such risk factor is diabetes. Diabetes leads to both microvascular and macrovascular disease, including coronary artery disease. It is well established that the prevalence of diabetes in SMI exceeds that in the general population (up to a 50% increase).^30^ Possible contributing factors to this increased prevalence include the effects of antipsychotic medication, socioeconomic determinants, and inherited risk. In patients with SMI, a diagnosis of diabetes confers an increased risk of death.^31^ Interestingly, when considered as a single group, patients in London with SMI and diabetes have lower (age‐adjusted) hemoglobin A1c levels than the non‐SMI reference population with diabetes.^32^, ^33^ There are also inequalities in diabetic control related to race and ethnicity: in an analysis of primary care data from the United Kingdom, patients of (self‐ascribed) South Asian and East Asian ethnicity had higher hemoglobin A1c levels compared with those of White British ethnicity.^33^

In addition to diabetes, SMI is associated with a higher prevalence of other modifiable cardiovascular risk factors, such as obesity and sedentary lifestyle,^34^ dyslipidemia,^35^ and smoking. A greater proportion of patients with schizophrenia smoke; those who smoke do so more heavily than individuals without psychiatric disease.^36^, ^37^ Although rates or smoking in the general population has declined over the past 2 decades, this is not the case in patients with mental illness.^38^ Given the high rates of both smoking and cardiorespiratory mortality in SMI, it is reasonable to consider that smoking is a potential key driver of CVD in SMI.^5^

It is notable that cardiovascular risk screening tools in routine practice, such as the QRisk3 tool, may miss risk in younger individuals, even after inclusion of antipsychotic medication as a factor, and latterly cardiometabolic risk predictor tools, especially for people with first‐episode psychosis, have been developed to address this unmet need.^39^

## Pathways to CVD Health Care: Recognition of CVD in Patients With SMI

The importance of the ECG in risk stratification of patients with SMI is highlighted by Danish primary care data suggesting that ECG abnormalities in patients with SMI confer a proportionally greater risk of cardiovascular death than controls.^40^ It is likely that a proportion of short‐term cardiac events go unreported and/or undiagnosed in patients with SMI. Retrospective analysis of ECGs at a Danish psychiatric hospital suggested that 75% of acute myocardial infarctions in psychiatric patients were or had been missed.^41^ Recognition of incident CVD is clearly of importance in patients with SMI: patients presenting with ST‐segment–elevation myocardial infarction (STEMI) had a longer duration of ischemic symptoms before undergoing primary angioplasty.^42^ Aside from ischemic heart disease, clinical studies have demonstrated increased rates of a range of cardiac abnormalities in patients with SMI, including structural issues, such as concentric cardiac remodeling (independent of age, race and ethnicity, and blood pressure),^43^ and electrophysiological pathological conditions, such as Brugada syndrome.^44^ Although antipsychotic medications are associated with prolongation of the QT interval, the underlying prevalence of long‐QT syndrome in patients with SMI is low.^45^

## Pathways to CVD Health Care: Revascularization

In the United Kingdom, ischemic heart disease and heart failure are managed through highly protocolized care pathways that are specifically designed to avoid delays in initiation of treatment. For example, when a patient is diagnosed with a STEMI, the first responder is empowered to deliver the patient directly to the regional Heart Attack Centre for emergency primary angioplasty, bypassing any local hospitals without these facilities to avoid delay in achieving reperfusion. Likewise, heart failure pathways are designed to ensure patients are under the care of a specialist heart failure multidisciplinary team to ensure rapid initiation and up‐titration of prognostic heart failure therapies.

Despite these systems, patients with SMI are much less likely to receive revascularization after an acute coronary syndrome (as suggested by a meta‐analysis of US studies),^46^ with the disparity more marked in patients with schizophrenia.^10^ Canadian data suggest that a mortality gap following acute myocardial infarction exists between patients with schizophrenia: patients with schizophrenia benefit as much as nonpsychiatric controls but have restricted access to revascularization.^47^ Furthermore, despite the commencement of highly protocolized heart attack pathways, the disparity in terms of revascularization rate between patients with and without SMI did not significantly improve between 1991 and 2014 in Scotland,^10^ or between 1996 and 2015 in Denmark.^48^

With particular regard to STEMI, US national data demonstrate that patients with SMI are less likely than controls to receive reperfusion therapy.^49^ Data from within the past decade from countries with established primary angioplasty networks suggest that patients with SMI and STEMI who undergo primary angioplasty have increased baseline risk versus controls (higher rates of smoking and longer duration of ischemic symptoms), but *once selected for invasive therapy* had similar procedural characteristics.^42^ They were, however, also less likely to receive prognostic secondary prevention medications at 1 year. Interestingly, rates of target vessel revascularization were unchanged across all durations of follow‐up. This may suggest 2 points: First, once selected for invasive therapy, angioplasty is likely to be technically successful with durable procedural results; and second, once patients are within the system, they are considered to be appropriate for future invasive approaches.

Given that there is retrospective US data to suggest that Black and Hispanic men with STEMI complicated by cardiogenic shock have worse outcomes than White men (as did women of any racial or ethnic group),^50^ it is important to identify whether racial and ethnic (and sex) disparities that are apparent in the care of the general population also have an effect in patients with SMI. The counterpoint to this is found in UK outcomes data, where no similar disparities have been demonstrated in the general population with acute coronary syndrome, but this has not been explored in patients with SMI.^51^, ^52^

## Secondary Prevention of CVD in Patients With SMI

The mortality gap from CVD between patients with SMI versus nonpsychiatric controls persists following a diagnosis of myocardial infarction or stroke.^9^ Following an event, patients with SMI are less likely to receive prognostic therapies, such as appropriate medication (aspirin or β‐blockers).^9^ It is also important to recognize that patients with SMI from underrepresented racial and ethnic groups also have less access to evidence‐based therapies for their psychiatric disease.^53^ Clozapine is the gold standard antipsychotic drug for resistant schizophrenia (albeit with significant adverse metabolic effects, which are most marked in patients of non‐White ethnicity).^54^ Patients with schizophrenia of Black ethnicity are less likely to be prescribed clozapine.^55^, ^56^ Whether this applies in CVD secondary prevention is not known, but antipsychotic use is associated with greater adherence to cardiac medications.^57^

Given this lack of access to therapies, it is perhaps unsurprising that patients with SMI have not derived the same decrease in mortality seen in the general population. It should go without saying that none of this is the fault of patients with SMI, but perhaps this is a message that cardiologists need to hear aloud and proactively discuss with their psychiatry colleagues. Health care professionals are more likely than psychiatrists to have a negative attitude toward patients with SMI.^58^ It is presumed that psychiatrists do not have an equally negative view of patients experiencing lobar pneumonia or ischemic gut.

## Strategies to Investigate the Interaction Between SMI, CVD, and Race and Ethnicity

As discussed above, patients with SMI have reduced access to revascularization, even in highly protocolized STEMI pathway. This is associated with poor outcomes. To identify exactly why this is will require analysis in racially and ethnically diverse populations who have a combination of both a high proportion of urbanicity and well‐established STEMI pathways with large patient volumes. It is for these reasons that London (which has an established STEMI pathway since 2005 and where health care is socialized) is an ideal population to study. London is much more racially and ethnically diverse than some other populations in the current evidence base. This diversity is vital to identify the role of race and ethnicity in outcomes for patients with SMI.

Addressing a problem as complex as the interaction between SMI and CVD necessarily requires a multifaceted approach. Strategies to enhance disease prevention, identification, and evidence‐based treatment initiation for those with incident CVD are urgently needed. Education on the enhanced risk of CVD in people with SMI and the potential for clinician bias should be embedded in our teaching. Linkage of psychiatric and cardiology observational data from electronic health records affords an opportunity to deploy data science solutions to identify patients at risk of CVD and/or those patients with CVD who are not yet receiving optimal medical therapy. Coworking with mental health colleagues to plan reasonable adjustments where active symptoms of mental illness impede access or uptake of standard treatment pathways should be routine and early. At the heart of the matter, strategies must be in place to ensure that advances in medical treatment and technology are made available to and enjoyed by patients with SMI.

Another solution is to actively involve cardiologists in the care of patients with SMI, to afford the opportunity to actively seek out and treat cardiovascular risk factors and CVD, while at the same time providing appropriate management of cardiovascular complications of antipsychotic therapy.^7^ This would be consistent with the “outreach” approach increasingly being adopted by cardiologists in recent years (eg, subspecialties such cardio‐oncology and maternal medicine) and should be offered in both the hospital and the community.

In summary, patients with SMI, which is more frequent in Black and other underrepresented racial and ethnic groups in Western societies, are at increased risk of CVD and have inadequate access to evidence‐based therapies. Given the existence of such highly effective therapies, the increased burden of CVD and cardiovascular death that is seen in the population with SMI would be considered as “avoidable” in the general population. Strategies must be urgently put in place to abolish this gap in effective health care.

## Sources of Funding

This work was supported by the Maudsley Charity, the NIHR Applied Research Consortium, and the King's British Heart Foundation Centre of Research Excellence Grant (RE/18/2/34213).

## Disclosures

None.
